# European nurses’ attitudes towards assisted death: A scoping review

**DOI:** 10.1177/09697330251403140

**Published:** 2026-01-21

**Authors:** Marita Nordhaug, Heidi Jerpseth, Katrine Staats

**Affiliations:** 1 Faculty of Health Sciences, 158935 Oslo Metropolitan University, Oslo, Norway

**Keywords:** assisted death, euthanasia, assisted suicide, nurses, attitudes, Europe, scoping review

## Abstract

Assisted death, which encompasses euthanasia and assisted suicide, remains a contentious ethical and legal issue across Europe. As frontline healthcare professionals, nurses are uniquely positioned to interpret and respond to patients’ suffering as well as their complex requests for assisted death. This scoping review explores European nurses’ attitudes towards assisted death, examining the factors that shape their views. A systematic literature search was conducted in six databases, which was complemented by manual searches yielding 20 studies from various European countries. Thematic groups identified in the review include: (1) Legal and organisational conditions, (2) ethical tensions and moral reasoning, (3) nurses’ roles and responsibilities and (4) individual and professional characteristics. The findings highlight significant variations in attitudes towards assisted death, which are influenced by legal, ethical, cultural and organisational conditions. In those European countries where assisted death has been legalised, nurses have expressed a strong desire to be more involved in decision-making processes, reflecting their proximity to patients and their critical role in interdisciplinary teams. Conversely, in countries without legal frameworks for assisted dying, nurses often reported uncertainty and ethical quandaries when navigating patients’ requests for assisted death. Key factors that shaped these attitudes include demographic characteristics, religion, education, professional experience and work environment. Ethical tensions were identified between caring perspectives and principle-based concerns, and between respecting patient autonomy and adhering to the principle of non-maleficence. Additionally, differences in attitudes towards euthanasia and assisted suicide were linked to ethical distinctions between actively causing death and allowing death to occur. This scoping review underscores the need for enhanced training in communication and ethical competence, as well as the greater involvement of nurses in policy discussions and decision-making processes. The nursing profession can enhance its capacity to manage the ethical complexities of assisted death, ensuring that decisions are both ethically sound and patient-centred.

## Introduction

Globally, the topic of assisted death has been a subject of intense debate for decades within medical, ethical and legal domains.^1,2^ Its acceptance or rejection often hinges on personal, cultural or religious beliefs.^3,4^ Assisted death encompasses both euthanasia and assisted suicide, which are medical procedures carried out according to specific criteria in countries where it has been legalised.^5^ Euthanasia is defined as an act undertaken by a doctor or specialist nurse to intentionally end a person’s life at his or her request.^5,6^ Assisted suicide is defined as the act of helping another person to voluntarily end their own life, typically by providing or facilitating access to means that can be used to cause death.^5^

Nurses are key frontline providers in healthcare, maintaining close contact with patients and families, and collaborating with physicians and other professionals. Their partnership with patients is vital for high-quality care. As such, nurses’ views on complex end-of-life issues can influence policy, especially in regions that are considering legal changes. Despite their central role, nurses have been underrepresented in assisted death debates.^7–9^ This is a missed opportunity, as their unique insights and frontline experience could greatly enrich these discussions.

Understanding nurses’ perspectives can facilitate better communication and stronger relationships between patients and healthcare providers. A scoping review is important because it provides a comprehensive overview of current knowledge, identifies trends and gaps in the literature and lays the foundation for future studies and practical initiatives. This contributes to strengthening nurses’ ability to provide ethical and professional care in the face of complex and sensitive issues such as assisted death.

## Background

Across Europe, the legal status and societal acceptance of euthanasia and assisted suicide vary widely. The Netherlands, Belgium and Luxembourg have legalised these practices under strict conditions with detailed legal frameworks.^10,11^ In contrast, many countries maintain restrictive or prohibitive policies. A west-east divide exists, with Western European nations, especially those with legal access, showing greater acceptance than Eastern ones.^10,12^ These differences stem from ethical and contextual factors such as patient autonomy, sanctity of life, fears of misuse and religious or cultural opposition.^13^ For nurses, these factors significantly shape their attitudes, often creating ethical dilemmas when institutional policies conflict with patient wishes. From a European human rights perspective, hospitals with restrictive policies must inform patients of alternatives if their requests conflict with institutional ethics.^14^ Legal disparities have also led to ‘patient migration’, where individuals travel to more permissive countries for assisted dying.^15^

Given the complex and evolving landscape, the following section provides an overview of country-specific regulations. This overview serves as a foundation for exploring and understanding nurses’ perspective and attitudes towards assisted death. Table 1 summarises the legal status of euthanasia and assisted suicide in selected European countries, including the year of legalisation and the scope of permitted practices.

**Table 1.** Legalisation of assisted death in Europe.

Countries in Europe that have legalised assisted death	Year of legalisation for assisted death	Euthanasia	Assisted suicide (termed either medically assisted suicide (MAS) or physician-assisted suicide (PAS))
Netherlands	2002	✓	✓
Belgium	2002	✓	✓
Luxembourg	2009	✓	✓
Switzerland	1946		✓
Germany	2015		✓
Spain	2021	✓	✓
Austria	2022		✓
Portugal^a^	2023	✓	✓

^a^As of 2025, the legislative process is ongoing, with recent approvals indicating that euthanasia may soon be fully legalised, under specific conditions.

In 2002, the Netherlands became the first European country to legalise euthanasia.^16^ The law allows euthanasia and physician-assisted suicide (PAS) for patients who are enduring unbearable suffering with no hope of improvement and requires a voluntary request and approval by two doctors. Belgium also legalised euthanasia in 2002, under similar conditions, later extending it in 2014 to include minors who are capable of discernment in hopeless, unbearable medical situations.^17^ In Switzerland, euthanasia is illegal, but PAS is permitted if not driven by selfish motives.^18^ Germany recently overturned its ban on assisted dying, whilst Austria continues to prohibit it.^19^ In most countries in Europe, both euthanasia and assisted suicide are illegal. In Norway, for example, a person who commits assisted dying will be punished with imprisonment ranging from 8 to 21 years.^20^ The legal aspect is reflected in the nursing ethical guidelines, where it is specified that a nurse may not, under any circumstances, contribute to euthanasia or assisted suicide.^21^

Assisted death remains a politically and ethically debated issue in healthcare. Legal status and societal acceptance vary across Europe, which is likely to be reflected in nurses’ attitudes. As demonstrated in Table 1, there are continuous changes in legislation across different countries regarding assisted death. It is therefore essential to present recent research that can provide insight into nurses’ attitudes in relation to the legal frameworks of their respective countries. Exploring these attitudes and their underlying factors is essential. According to Maio and Haddock,^22^ attitudes are shaped by cognitive, emotional and behavioural processes, which often interact in complex ways. For nurses, these attitudes influence patient care and interprofessional collaboration in assisted death cases. Although nurses must adhere to professional standards that may limit the scope for personal beliefs, they also need a deep understanding of the values that guide their profession.^23^ This includes balancing specialised knowledge and responsibilities with the unique needs of individual patients. Given nurses’ close patient relationships and central role in care teams, it is important to consider both the diversity of their attitudes and the shared influences across European contexts. Based on our knowledge, there are few studies that collectively examine European nurses’ attitudes towards assisted death. In 2020, a scoping review was published with the aim of exploring what is known about nurses’ perceptions and attitudes towards euthanasia.^24^ However, that study included nurses from around the world and focused solely on euthanasia, without addressing their attitudes towards assisted suicide. Over the past 5 years, several European countries have transitioned from prohibiting both euthanasia and assisted suicide to legalising either or both practices (see Table 1).

Understanding nurses’ perspectives may facilitate better communication and stronger relationships between patients and healthcare providers. A scoping review is therefore important, as it provides a comprehensive overview of current knowledge, identifies trends and gaps in literature and establishes a foundation for future research and practical initiatives. By summarising and synthesising nurses’ attitudes towards euthanasia, such a review can contribute to improving communication, enhancing the quality of care and supporting nurses in navigating their ethical and professional responsibilities.

This scoping review explores European nurses’ attitudes towards assisted death, examining the factors that shape their views. The aim of this scoping review is to provide an overview of how nurses’ attitudes towards assisted death are represented and discussed in the literature, and to identify conceptual patterns and gaps that may inform future research and ethical reflection.

## Method

### Design

To explore European nurses’ attitudes towards euthanasia, we employed a scoping review methodology, which is suitable for mapping evidence in a given field.^25^ As noted by Munn et al.,^26^ scoping reviews are effective for rapidly reviewing emerging or underexplored topics and identifying knowledge gaps. We followed Arksey and O’Malley’s five-step framework: (1) identifying the research question, (2) finding relevant studies, (3) selecting studies, (4) charting data and (5) summarising and reporting results.^27^ The review adhered to Joanna Briggs Institute (JBI) guidelines.^25^

### Identifying the research question

The research questions investigated in this scoping review were as follows:

(1) What are the attitudes towards assisted death among European nurses?
(2) What influences these attitudes?

### Identifying relevant studies

#### Search strategy

The systematic search for this scoping review was carried out twice – first in July 2022 and then an update search in July 2024. We utilised the following databases: Medline, Embase, Cinahl, PsychINFO, Web of Science and Scopus. To supplement the database searches, we also undertook a manual search with a particular focus on scanning reference lists of the included papers. This dual approach enabled us to follow replicable steps, thereby ensuring a more rigorous and comprehensive search.^28^ The search strategy contained both subject terms and text words describing the main concept in this scoping review: ‘euthanasia’, ‘nurse’ and ‘attitudes/perceptions’. We used the Boolean operator OR to combine search terms within the same concept, then linked the words from each concept using AND. This search was conducted under the guidance of a university head librarian. To ensure a baseline level of quality, only peer-reviewed studies with abstracts were included in the scoping review. Grey literature, non-peer-reviewed studies and studies without abstract were excluded to maintain consistency and ensure that all included studies adhered to established standards of scientific rigour. Similarly, studies without English translation were excluded to ensure accurate analysis and interpretation of their content, as the authors are proficient in English and wanted to avoid risks of misinterpretations due to language barriers. We also searched for registered or ongoing studies of ‘nurses’ attitudes towards euthanasia’ in PROSPERO & DARE (International Prospective Register of Systematic Reviews) for registered or ongoing systematic reviews related to ‘nurses’ attitudes towards euthanasia’, to ensure our review did not duplicate existing work, revealing no study conducted recently or likely to be conducted. The search strategy is included in Table 2.

**Table 2.** Search terms and search results per 05.07.2022.

No	Search terms	Medline	Embase	Cinahl	PsychINFO	Web of Science	Scopus
1	(health/medical personnel/staff/provider/worker/professional/clinician/practitioner) OR (nurse*/nursing*)	1250474	2431687	1160914	374680		
2	(attitude*) OR (perception) OR (perceiv*) OR (exp health personnel attitude) OR (attitude to death)	764293	9708881	575032	765205		
3	(euthanasia/active/voluntary/suicide/assisted) OR (‘right to die’) OR (‘death with dignity’) OR (euthanas*)	32181	32138	13099	3636		
1 and 2 and 3	Total 9723	2243	2036	1518	506	578	2842
Update search per 10.07.2024	Total 764	121	192	131	45	115	160

All screening steps were based on the following inclusion and exclusion criteria:

**Textbox 1.** Inclusion and exclusion criteria

**Inclusion criteria**

• Published after January 2002 in English language.
• Studies focussing on nurses as the primary population of interest.
• Having Europe as the research context.
• Peer-reviewed studies with abstracts.

**Exclusion criteria**

• Studies without an English translation.
• Research was conducted from the perspectives of patients or other healthcare professionals.
• Research was conducted outside Europe.
• Theoretical articles, letters, book reviews, commentaries and unpublished papers were excluded.

#### Charting the data

The data were systematically charted in customised data charting form, which encompassed key information that was relevant to our research question. The form was designed to ensure consistency and thoroughness in capturing essential data across all included studies. The data chart included details such as the studies’ aims, country, design, participant characteristics, sample size and key findings related to the research questions (see Table 2).

The development of the data charting form followed an iterative process adhered to the JBI recommendations for scoping reviews and the framework outlined by Levac et al.^29^ This process allowed for ongoing refinement of the form to ensure it aligned with the objectives of the review and the complexities of the selected studies. Each study was carefully reviewed, and data were extracted systematically to reduce bias and maintain consistency across the charting process.^24^

#### Collating, summarising and reporting the results

Building on the systematic charting process outlined above, the extracted data were carefully reviewed and analysed to identify patterns, similarities and differences relevant to the research questions. An interactive, inductive approach was used to organise that data into thematic groups. Initially, the authors independently familiarised themselves with the charted data in Table 3 and noted recurring concepts and key ideas. Through a collaborative process, similar data points were grouped into preliminary codes, which were then refined into broader thematic groups. These groups were developed to reflect the key findings from the included studies and to address the study’s research aims. Examples of resulting thematic groups include ‘Education, professional experiences and work environments’ and ‘General attitudes, involvement and competence’. To ensure the validity and coherence of the thematic structure, the authors engaged in multiple rounds of discussion, refining and consolidating thematic groups until consensus was reached.

**Table 3.** Study information (author, year, country, aim, design, sample size and participant characteristics) and key findings.

Study information				Key findings	
Author, year, country	Aim	Design	Sample size and participant characteristics	What are the attitudes towards assisted death among European nurses?	What influences these attitudes?
Bendiane et al. (2007)	To assess French district nurses’ opinions on euthanasia	Anonymous telephone survey design	602 nurses	About 65% of district nurses supported legalising euthanasia	Attitudes were influenced by gender, age, religion and professional experience. Men and nurses under the age of 50 were more supportive of euthanasia, whilst those with strong theistic beliefs were less so. Attendance at palliative care or pain management conferences correlated with lower support, whereas frequent end-of-life discussions with patients increased support. Nurses sceptical of always following surrogate decisions were also less supportive
France			District nurses delivering home care with at least 1 year of professional experience		
Bendiane et al. (2009)	To investigate hospital nurses’ opinions on both legalisations (euthanasia and PAS), including personal conceptions of euthanasia and working conditions and organisations	Anonymous telephone survey design	1502 nurses	Overall, 48% of nurses supported legalising euthanasia and 29% supported PAS. Of them, 28% strongly agreed it should be legal for terminal or incurable patients, 11% strongly disagreed and 15% were neutral	Support for euthanasia and PAS was lower among nurses with strong religiosity, palliative care training or confidence in end-of-life care. Night-shift nurses showed more support, possibly due to stress and job dissatisfaction. Support was weaker in palliative and intensive care units, but stronger in units with more staff and no religious affiliations. Cultural values favouring autonomy also played a role, with less trained and experienced nurses showing more support for euthanasia than for PAS.
France			Working in hospitals and frequently confronted with end-of-life issues		
Brzostek et al. (2008) Poland	To explore the perception of palliative care and euthanasia by nurses	Quantitative explorative design utilising questionnaire	458 nurses	Most nurses viewed euthanasia and palliative care as incompatible, were less willing to accept it for relatives, but more open to it for themselves	Nurses’ attitudes were shaped by their philosophy of life, experience, knowledge, religious beliefs and views on law and professional ethics. Whilst experienced nurses valued legal guidance more, the nurses often saw it as less relevant in end-of-life care. Personal beliefs outweighed adherence to the professional deontological code
			206 recent graduates. Six males and the rest female. Mean age ±28 years		
			252 experienced nurses. All female. Mean age ±47 years. Majority worked in hospital		
De Hert et al. (2015) Belgium	To provide insight into the attitudes and actions taken by psychiatric nurses when confronted with a patient euthanasia request based on unbearable mental suffering (UMS)	Quantitative descriptive design utilising a questionnaire	627 nurses	Most participants did not oppose euthanasia based on UMS. Few supported limiting it to physical suffering, and 19 found it ethically unacceptable	Nurses’ attitudes towards euthanasia varied by ward type and patient diagnosis. Those in acute settings and working with psychotic or personality disorder patients were more supportive of euthanasia, especially for UMS, than those in long-term care or addiction units. No significant links were found with gender, education, experience or views on capacity or suicide. Many psychiatric nurses reported lacking the skills and tools to assess euthanasia requests, particularly regarding capacity of will. Attitudes may have been shaped by care models, rehabilitation in long-term care fostered hope and acute settings exposed nurses to more severe cases and visible treatment effects
			All nurses aged 35 years or older.		
			>50% worked for more than 10 years. Most were female		
Demedts et al. (2018) Belgium	To determine the attitudes, role and knowledge of mental health nurses regarding UMS euthanasia	Cross-sectional design	133 nurses	Most mental health nurses supported UMS euthanasia. Three quarters believed that psychiatric patients could make well-considered requests, and nearly all agreed that they could experience medical hopelessness, although views varied by diagnosis. Two-thirds said that recognising hopelessness does not undermine care or hope. Although most were comfortable discussing UMS euthanasia with patients, many found it harder to do so with physicians	Nurses’ attitudes towards euthanasia were shaped by legal, policy, clinical and educational factors, as well as patient diagnosis and age. They believed that mental suffering could be unbearable and that psychiatric patients may be competent to request UMS euthanasia. Nurses recognised their role but sought clearer guidelines and more training. Their concerns centred on decision-making capacity, the irreversibility of suffering and legal uncertainties
			Mental health nurses with >10 years of experience from both acute and treatment wards. Most nurses were female. Average age >35 years and with a religious faith		
Dörmann et al. (2023)	To assess the views and attitudes of nurses from different care settings regarding assisted suicide	Qualitative design utilising grounded theory	20 nurses	Nurses showed ambivalence towards assisted suicide but stressed the need for support in forming personal views. They sought more knowledge to be able to act professionally in vulnerable situations and educate others. This enabled them to tolerate patients’ wishes to die while offering consistent presence and care, regardless of personal beliefs	Nurses’ main concern in assisted suicide was understanding the desire to die, which they assessed individually due to their wavering attitudes and limited experience. Developing this understanding helped them to find their stance, with personal life experiences strongly shaping their views
Germany			14 females and 6 males from different care settings. Average of 44.8 years of age and 21.2 years of professional experience		
Francke et al. (2016)	To give insights into Dutch nursing staff’s attitudes and involvement regarding euthanasia	Nationwide existent survey	587 nurses	Most nurses valued being involved in euthanasia decisions. Nearly 70% believed that physicians should consult nurses who regularly care for the patient, and even more said that nurses should be included when deciding to administer lethal drugs	Nurses’ attitudes towards euthanasia were shaped by religion, workplace and experience. Religious nurses and those in academic hospitals, general hospitals or home care were more likely to support physician–nurse discussions on euthanasia. Nurses in academic hospitals were also more likely to support nurse-administered euthanasia. Being a registered nurse, working in hospitals or nursing homes, caring for terminal patients or being part of palliative care teams were linked to actual involvement in euthanasia decisions
The Netherlands			Registered nurses (RNs) and certified nursing assistants (CNAs). 94% female. Mean age of 47 years and an average of 22 years of nursing experience		
Gielen et al. (2011)	To assess the influence of religion and world view on palliative care nurses’ attitudes towards euthanasia	Quantitative design utilising a secondary analysis	327 nurses	Nurses with strong religious or ideological beliefs – especially those who frequently attended services or believed in a God who intervenes in human life – were less likely to support euthanasia. Among palliative care nurses in Flanders, ideology was the strongest influencing factor	Nurses’ attitudes towards euthanasia were strongly shaped by religion and worldview, particularly mass attendance and the content of belief – the latter being most influential. In Western Europe, where nurses often form personal religious identities, these beliefs significantly impacted their views
Belgium			Flemish palliative care nurses from different clusters (atheist/agnostics, doubters, church-going, religious but not church-going and devout church-going)		
Hol,et al. (2022)	To investigate Norwegian clinical nurses’ attitudes towards assisted dying	Quantitative and exploratory design utilising the Norwegian Bioethics Attitude Survey	205 nurses	Nurses in this study were less supportive of legalising assisted dying than the general Norwegian population. About half supported legalisation, noting that requests – especially in pulmonary wards – were common	Nurses’ attitudes towards assisted dying varied by age, education, experience and ward type. Younger nurses were more supportive, whilst those with postgraduate training – especially in palliative care – were less so, focussing on relieving suffering. Nurses in pulmonary wards, who faced frequent requests and ethical challenges, tended to be more supportive
Norway			Registered nurses from four hospitals and one home care district. All performed bedside care. 91% female. 38% were aged 20–29 years. 42% with postgraduate education		
Hol et al. (2023)	To explore Norwegian nurses’ perception of dying requests from terminally ill patients	Qualitative research design	15 nurses	Nearly all participants expressed uncertainty about how to respond to assisted dying requests	Nurses’ attitudes were shaped by ethical challenges, feelings of unpreparedness, moral uncertainty and distress – especially in settings where euthanasia is taboo. Chaplains offered key support. Nurses called for open, professional discussions beyond legal debates. Confidentiality concerns and limited knowledge contributed to moral distress, highlighting the need for better communication skills
Norway			Registered nurses. 12 females and 3 males. Worked full-time bedside in hospital or home care and had received an assisted dying request at some point		
Inghelbrecht et al. (2009a)	To investigate nurses’ attitudes towards different ELDs, and towards their role in those decisions	Cross-sectional design	3321 nurses	Nearly all nurses (96%) agreed that terminally ill patients should receive pain-relief drugs, even if these may hasten death	Nurses’ attitudes towards euthanasia were shaped by religion, workplace, education and experience. Religious, especially Catholic, nurses showed lower acceptance of euthanasia and patient autonomy. Home care and nursing home nurses were less supportive of continuous deep sedation (CDS) than hospital nurses. Palliative care training increased support for withholding or withdrawing life-sustaining treatment. Higher-educated nurses were more prepared to administer drugs for euthanasia and CDS.
Belgium	To aim to detect differences in attitudes between groups of nurses based on their social-demographic and work-related characteristics		88% female. 77% aged >36 years. 63% Catholic and 52% worked in a hospital. 90% had cared for terminally ill patients and 51% had experiences with ELDs in the last 12 months		
Inghelbrecht et al. (2009b)	To explore nurses’ nationwide attitudes towards euthanasia and their role in it	Cross-sectional design	3327 nurses	92% of nurses supported euthanasia for terminal patients in extreme, uncontrollable distress. Over half (57%) accepted life-ending acts without patient request in cases of unbearable suffering and incapacity. Whilst 70% believed that good palliative care could prevent most euthanasia requests, 26% saw terminal sedation as a better alternative	Nurses’ attitudes towards euthanasia were shaped by religion, age, experience and role. Religious nurses and those who saw religion as professionally important were more opposed. Older nurses were more supportive of life-ending without request and favoured palliative care or sedation as alternatives. Head nurses and managers were less supportive of life-ending without patient consent
Belgium	To examine how these attitudes relate to socio-demographic and work-related characteristics		88% females. 63% Catholic. 51% with a baccalaureate degree. 52% worked in a hospital. 90% had cared for terminally ill patients and 51% had experiences with ELDs in the last 12 months		
Ortega-Galán et al. (2023)	To learn about the attitudes of nurses regarding euthanasia and anxiety about death in relation to age and work experience	Observational descriptive study	518 nurses	Younger, less experienced and part-time nurses showed the most positive attitudes towards euthanasia and held more utilitarian views. Support declined with age and experience	Nurses’ attitudes towards euthanasia were shaped by age and experience. Younger, less experienced and part-time staff tended to be more supportive and held utilitarian views. Support declined with age and experience. Some professionals showed a gap between positive views and reluctance to participate
Spain			76.6% female and 23.4% male. Mean age of 44.75 years. 30.7% worked in primary care, 27.6% in inpatient units, and the remaining in critical care units, mental health and outpatient consultations		
Velasco Sanz et al. (2022)	To find out the opinions and attitudes of nurses regarding the regulation of euthanasia and MAS	Cross-sectional descriptive design, survey	489 nurses	Nurses – particularly those aged over 50, with over 30 years of experience, working in the public sector, and who were non-religious – largely supported the regulation of euthanasia and MAS. There was stronger consensus on their role in deliberation and patient support during assisted dying than in drug administration, especially among those with postgraduate training in palliative care and bioethics. Support was greater for nurse involvement in MAS than in euthanasia, with both seen as applicable by doctors and nurses	Nurses’ views on euthanasia were shaped by religion, gender, palliative care quality, legality and patient autonomy. Older nurses with extensive experience and postgraduate training in bioethics and palliative care tended to be more supportive. Conscientious objection was low, influenced by age, experience, care models and religious beliefs. Religion affected 77.7% of attitudes, underscoring the need to balance ideological freedom with patients’ legal rights
Spain			Registered nurses, 86.3% women aged between 31 and 40 years. 25.8% had more than 20 years of work experience. 78.9% working in the public health system. 52.8% considered themselves to be religious believers		
Tamayo-Velazquez et al. (2012)	To explore the knowledge, opinions and experience of nurses in relation to euthanasia and PAS	Descriptive, cross-sectional design	390 nurses	22.6% of nurses believed that euthanasia had been performed by a healthcare professional in the past 5 years. Nationally, 76% supported its legalisation, and 54% said they would perform it if legal. Half (50.3%) believed that terminally ill patients should have the right to euthanasia, whilst 35.6% opposed it	Nurses’ attitudes towards euthanasia were shaped by gender, age and legal knowledge. Male nurses reported more requests for euthanasia, and those aged under 45 were more aware of its legal status. Half believed that quality palliative care reduces such requests, although 28.3% disagreed. Many nurses struggled to identify appropriate cases, thereby highlighting the need for better education
Spain			Nurses in both primary and specialist care. 31.9% male and 68.1% female. Mean age of 46.18 years. In the past year, 78.8% had cared for at least one terminal patient, and 32.6% had cared for more than ten		
Terkamo-Moisio et al. (2015)	To elucidate nurses’ rarely explored perceptions of euthanasia and its legalisation	Qualitative descriptive design	17 nurses	Most nurses believed that euthanasia was likely to be legalised in Finland in the near future. Their perceptions were shaped by ethical codes and personal experiences, often leading to conflicting views and avoidance of the topic. Whilst some saw euthanasia as justified in certain cases, there was a clear need for more open discussion and reliable information	Nurses’ attitudes towards euthanasia were shaped by ethical codes and personal experiences. Making it mandatory was seen as potentially traumatic for healthcare professionals. Many wondered whether denying euthanasia would mean leaving patients to suffer alone. There was a clear need for more education, with eight identified factors influencing perceptions, including the patient’s will
Finland			Registered nurses (both female and male). Ages ranged from 28 to 64 years. Worked in primary care hospitals. Length of experience varied from 4 to 36 years		
Terkamo-Moisio et al. (2017)	To explore the attitudes of Finnish registered nurses towards euthanasia	Cross-sectional web-based design	1003 nurses	Most nurses in the study would accept euthanasia in Finnish healthcare, with 68.7% expecting legalisation. However, 37.8% were unsure of its benefits and 37% feared potential misuse. Still, 80% viewed it as a humane option, 81.9% supported the right to choose death and 77.4% would consider it for themselves in certain situations	Nurses’ attitudes towards euthanasia were shaped by factors such as marital and parental status, religion, religiosity, experience, education, age and self-assessed expertise in end-of-life care. Religiosity showed the strongest correlation with acceptance, followed by views on legalisation and communication. Younger, less experienced nurses were most supportive of euthanasia and its legalisation, whilst more experienced nurses favoured open discussion. Belief in self-determination and control over one’s death also influenced attitudes
Finland	To explore the connection between demographic and work-related factors and nurses’ euthanasia attitudes		Mean age 39.54 years. Average work experience of 13.16 years. Most worked in specialised healthcare and encountered dying patients monthly or less frequently		
Terkamo-Moisio et al. (2019)	To describe Finnish nurses’ attitudes towards their potential role in the euthanasia process	Cross-sectional web-based design	1003 nurses	Most nurses (85.2%) felt that their views should be considered in euthanasia decisions, and 73.7% supported active involvement. Preparing patients was seen as part of their role by 72%, while over half agreed with handling euthanatics (61.4%) and inserting cannulas (69.2%). A large majority (88.6%) believed nurses should be present if the patient wished, although only 32.9% supported mandatory participation. Still, 74.7% would personally take part if euthanasia were legal	Nurses’ attitudes towards euthanasia were shaped by age, experience, education, family status, religiosity and self-assessed expertise. Older and more experienced nurses were less supportive of active involvement, whilst younger, less experienced, unmarried and childless nurses showed greater support, especially for handling euthanatics. Higher education increased support for technical tasks and patient preparation. Religious beliefs correlated with lower support, whereas non-religious nurses favoured active roles. Interestingly, those who were confident in end-of-life care were less likely to support handling euthanatics
Finland			93% were females. Mean age 39.54 years. Average work experience of 13.16 years. Most worked in specialised healthcare and encountered dying patients monthly or less frequently. 50.8% of the nurses were religious		
Van Bruchem-Van De Scheur,et al. (2008)	To investigate the role, perceptions, responsibilities and problems of nurses in medical end-of-life decisions in order to advise the Dutch government on legislation and policymaking	Mixed-method design	1179 nurses	Fewer than half of the nurses (45%) were willing to serve on euthanasia review committees. A majority (58.2%) felt it was excessive to require physicians to consult nurses in decision-making. Most nurses rejected preparing euthanatics (62.9%) and inserting infusion needles (54.1%) as part of their role	Nurses’ attitudes towards euthanasia varied by work sector, particularly regarding preparative tasks. Sector-specific skills and practices significantly shaped these views
The Netherlands			The largest proportion worked in hospitals, followed by home care and nursing homes. 87.9% were female. Mean age of 40.6 years. Mean experience of working as a nurse of 16.5 years		
Van Humbeeck et al. (2022)	To explore the legal understanding and attitudes of nurses and physicians regarding euthanasia within the context of tiredness of life (ToL) in older people	Quantitative	151 nurses	Nurses showed greater understanding than physicians regarding euthanasia requests based on tiredness of life, even without severe illness. They were more accepting of such requests when patients felt they had lived a fulfilled life, and agreed that unbearable suffering could include existential fatigue	Nurses’ close relationships with older patients often made them more supportive of families than physicians. In cases of reversible, non-terminal illness, 40.4% of nurses believed that euthanasia has legal grounds. This gap reflects how caregiving roles shaped nurses’ perceptions, sometimes diverging from legal understanding
Belgium		A survey with four case vignettes	Nurses employed in one home care organisation, two hospitals and one nursing home group. About half worked in acute care and half in chronic care		

## Results

### Search outcomes

The primary search in 2022 resulted in 9732 records. After de-duplication, which was performed both automatically and manually in EndNote, 5075 records remained. A further 1716 were removed for other reasons. 3359 records were screened based on title and abstract, after which we ended up with 155 articles. Records were uploaded to Rayyan, which is a web-based systematic review program,^30^ with ‘blind-on’ to manage the review process. The first and third authors concurrently and independently applied the inclusion and exclusion criteria in Rayyan and identified those articles that met the research aim by reading titles and abstracts. The discrepancies were resolved with the second author, and 27 articles were read in full text. Finally, 16 articles were included. An update search was carried out in 2024 and added 5 more articles, which made a total of 20 articles to be included in this scoping review.

**Figure 1.**
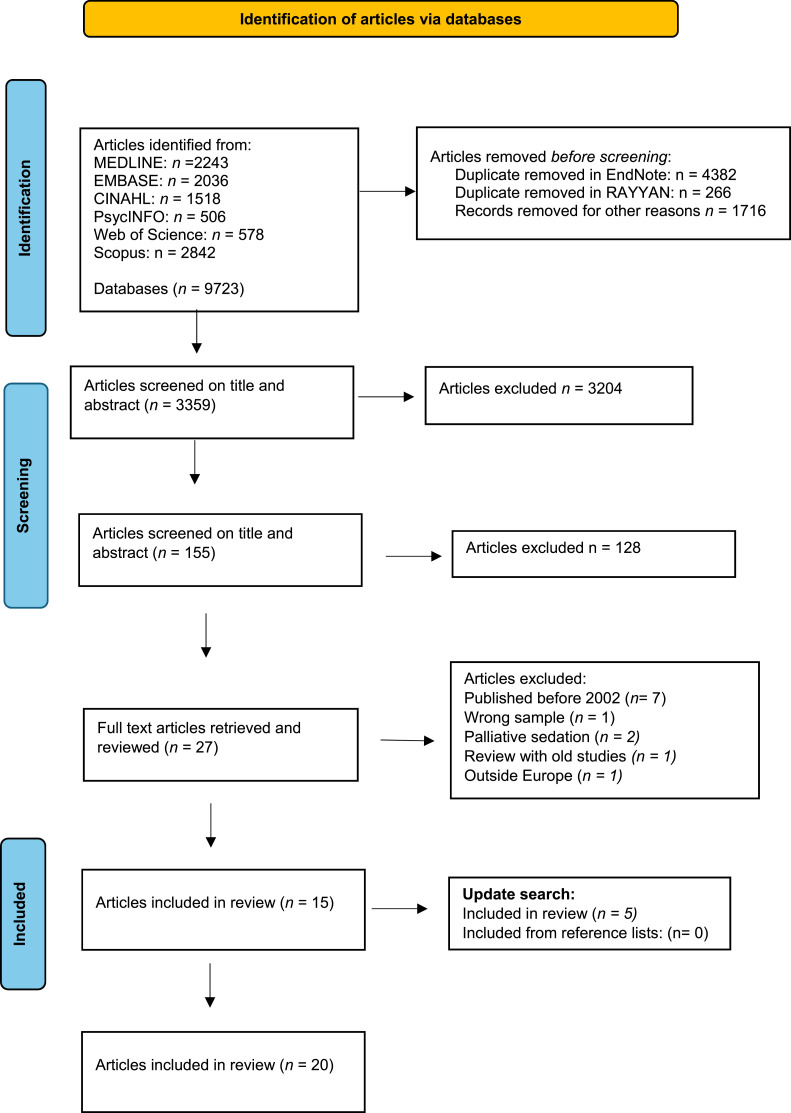
PRISMA-ScR flow diagram.^31^

The complete selection process is shown in the PRISMA-ScR flow diagram (Figure 1).

### Study characteristics

In total, 20 studies about attitudes towards euthanasia among European nurses were identified from the records. The studies were conducted in Norway (*n* = 2),^9,32^ Belgium (*n* = 6),^33–38^ Spain (*n* = 3),^39–41^ Germany (*n* = 1),^42^ France (*n* = 2),^43–44^ Poland (*n* = 1),^45^ the Netherlands (*n* = 2)^46,47^ and Finland (*n* = 3).^8,48,49^

The 20 studies included informants from various layers within healthcare, such as hospitals, nursing homes and home care services. Of these, 15 studies primarily focused on hospital settings and five concentrated on nurses working in municipal health services.^39–41,43,46^

In the 20 studies identified, a total of 158.74 nurses participated as informants, either as informants in qualitative studies (*n* = 3, 0.33%) or informants in quantitative studies (*n* = 17, 99.7%). They had an average age of ˃40 years, with a substantial amount of work experience, and most nurses frequently encountered dying patients. In relation to the large proportion of quantitative studies, there is a distribution of studies utilising surveys,^32,34,39,43–46^ one survey with case vignettes,^33^ cross-sectional designs^35,37,38,40,41,8,49^ and quantitative secondary analysis.^36^ Three studies had a qualitative design^9,42,48^ and one a mixed-method approach.^47^

### Thematic groups

In addressing the study’s first aim – that is, identifying the attitudes towards assisted death – two thematic groups emerged from the analysis: *General attitudes, involvement and competence* and *Ethical perspectives.* As for the second aim of the study – that is, identifying the factors that influence these attitudes – four thematic groups were identified: *Demographics and gender; Religion, culture and ethical perspectives; Education, professional experience and work environments* and *The impact of patients’ characteristics.*

### European nurses’ attitudes towards assisted death

#### General attitudes, involvement and competence

The legalisation status and practice of assisted dying vary widely across Europe. In countries where it remains illegal, most nurses support legal regulation of euthanasia, MAS and PAS.^32,43,48,8,49^ A Spanish survey found that 76% of nurses in primary and specialist care supported legalisation, with 54% willing to perform euthanasia if legal.^40^ Following the legalisation of MAS in Spain in 2021, a later study showed that 75.7% and 72.6% of nurses supported the legal regulation of euthanasia and MAS, respectively.^41^ In France, 48% of nurses supported the legalisation of euthanasia and 29% supported PAS.^44^ In Norway, Hol et al.^32^ found that 56% of nurses supported legalising PAS and 48% supported euthanasia.

Nurses expressed nuanced views on their involvement in euthanasia. In Finland, 74.7% said that they would participate if it were legal.^8^ In the Netherlands, most nurses felt that tasks such as inserting infusion needles and preparing euthanasic should not be their responsibility.^47^ Some findings reveal a personal dimension – for instance, Polish nurses were more open to assisted death for themselves than for close relatives.^45^ Another study showed that nurses expressed a strong desire to be more engaged in discussions and decision-making processes concerning euthanasia.^34^ This sentiment was especially prominent among nurses in countries where euthanasia is legal – for example, a significant majority of nurses in the Netherlands emphasised the importance of nurse participation in euthanasia-related decision-making processes.^46,47^

Francke et al.^46^ found that half of Dutch nurses believed that patients were more likely to request euthanasia from a nurse than a physician. Even in countries where it is not legal, such requests were common; in Norway, 58% of nurses working in hospitals and home care reported having received them.^32^ A Norwegian qualitative study also revealed that nurses often faced emotionally challenging situations when alone with patients requesting assisted death.^9^ Nurses expressed a strong desire for more guidance and competence in the context of assisted death, citing unclear roles and a lack of adequate training.^34,42^ They emphasised the need for increased competence, particularly in communicating with patients who request euthanasia.^9,42,44^ Nurses also emphasised the importance of addressing euthanasia in nursing education.^35^ These needs for more knowledge and competence in communication were relevant for nurses in countries both where euthanasia is permitted by law and where it is not allowed.

#### Ethical perspectives

Inghelbrecht et al.^37^ stress the importance of involving nurses in ethical discussions and acknowledging their perspectives, in countries where assisted death is legal. However, nurses often struggle with ethically complex decisions, including continuous deep sedation (CDS), which is seen by some as a more ethical alternative to euthanasia.^35^ Although nurses may serve on euthanasia review committees, their input is typically limited to medical aspects rather than the ethics of care.^47^ De Hert et al.^34^ and Demedts et al.^35^ emphasise the ethical sensitivity of euthanasia, particularly in cases of unbearable mental suffering (UMS), and advocate for comprehensive ethics training for nurses, especially in psychiatric care.

Ethical challenges around end-of-life decisions are also reported by nurses in countries where assisted dying is not legal. Nurses often struggle to balance their duty to relieve suffering with respecting patient autonomy.^32,42,48^ Hol et al.^32^ found that such dilemmas can lead to increased requests for assisted dying, fostering more positive attitudes. A Polish study^45^ highlighted the ethical conflict between euthanasia and core medical principles, viewing palliative care and euthanasia as incompatible. However, it also emphasised the need for nurses who work closely with suffering patients to develop ethical sensitivity to effectively alleviate distress.^45^

### What influences nurses’ attitudes towards assisted death?

#### Demographics and gender

Demographic factors play a notable role in shaping nurses’ attitudes towards assisted death. Nurses in the youngest age groups tend to have more positive attitudes towards euthanasia,^32,39,43,44,49^ whilst they were less likely to support PAS.^44^ In a Norwegian study, however, nurses under 30 years of age were more likely to support PAS than those older than 30.^32^ This contrasts with older nurses and those with more work experience, who exhibited varied and often more conservative attitudes.^41,49^ Additionally, gender differences were evident, with male nurses showing greater support for assisted death,^44^ as they received more requests for euthanasia than female nurses.^40^ For example, Bendiane et al.^43^ discovered that in France, a smaller proportion of female nurses compared to male nurses were in favour of legalising euthanasia, and the male nurses reported more frequent requests from patients for euthanasia compared to their female counterparts.

#### Religion, ethical perspectives and culture

Several studies showed that religion and personal beliefs – for example, nurses with strong religious convictions, such as believing in a god who masters destiny^43^ – were profoundly influential in shaping nurses’ attitudes towards euthanasia.^36,37,39,43,45,49^ In this context, it appears difficult for nurses to assist with both euthanasia and MAS. Another factor affecting attitudes towards euthanasia was the cultural environment. In societies where euthanasia remained a taboo subject, there was generally less support for its practice.^9^ In the Netherlands, nurses who indicated that their religious beliefs affected their view on end-of-life decisions were more likely to agree that physicians should discuss euthanasia requests with the nursing staff involved in the care.^46^

The nurses’ ethical perspectives played a significant role in shaping their attitudes^8,41,45,48,49^ – for example, nurses who strictly adhered to ethical guidelines tended to oppose euthanasia.^45^ In Poland, adhering to a professional deontological code influenced the attitudes of experienced nurses to a greater degree than recently graduated nurses, but to these nurses, having a philosophy of life had greater influence on their attitudes than their deontological guidelines.^45^ Respecting people’s ability and right to make their own decisions about life and death also seemed to influence the attitudes of many nurses. For instance, shortly before the legalisation of medically assisted suicide (MAS) in Spain, 81% of nurses stated that people have the right to decide how they want to live their lives and how they want to die.^41^ Cultural contexts further compounded these perspectives, suggesting that both personal and professional environments critically informed attitudes towards end-of-life care practices.^32^

#### Education, professional experiences and work environments

Studies show that nurses’ education, knowledge and experience influence their attitudes towards assisted death.^32,41,43–45^ Nurses with postgraduate education in palliative care or oncology (e.g. advanced degree or certifications) were generally less supportive of euthanasia,^31,34^ whilst those with broader higher education (e.g. bachelor’s degrees) or shorter, specific palliative care training programs tended to be more supportive and open to involvement.^46,49^ Bendiane et al.^43^ found no link between recent terminal care experience and attitudes, but noted that nurses attending palliative care or pain management conferences were less supportive of euthanasia.

De Hert et al.^34^ reported that Belgian psychiatric nurses felt unprepared to handle euthanasia requests, echoing Dörmann et al.,^42^ who found that German nurses needed more knowledge to act professionally and support patients, regardless of personal views.

Work environment also plays a role. Nurses working night shifts, in acute care or with high patient suffering were more likely to support euthanasia.^33,34,37,43,44^ Hol et al.^32^ noted that pulmonary ward nurses received more requests related to managing patient suffering.

#### The impact of patients’ characteristics

Nurses’ attitudes towards euthanasia are also influenced by their perceptions of patient factors. Doubts about the patient’s ability to make a voluntary and well-considered decision,^35^ respect for life and a belief in the efficacy of palliative care contribute to less supportive attitudes.^34,35,47^ For instance, Bendiane et al.^43^ found that nurses in France who systematically or frequently discussed end-of-life issues with terminal patients were more likely to support the legalisation of euthanasia. In a Spanish study, half of the nurses (50.3%) thought that legally capable, terminally ill patients should have the right to assisted dying.^40^ Nurses who questioned a patient’s decision-making capacity or who held strong convictions about the sanctity of life were less likely to endorse euthanasia.^35^ In Belgium, for instance, most nurses accepted euthanasia for terminally ill patients experiencing uncontrolled pain and distress.^37^ In a national phone survey among 602 French district nurses, Bendiane et al.^43^ found that two-thirds agreed that euthanasia should be legalised for patients with a terminal illness or an incurable condition. In a cross-sectional survey of psychiatric nurses in Belgium, a vast majority believed that euthanasia was justified in cases of unbearable mental suffering (UMS), and that patients with psychiatric illnesses could make well-considered requests for euthanasia due to their UMS, viewing this as distinct from their disease.^35^ Moreover, those who had confidence in the ability of palliative care to effectively manage end-of-life symptoms were more inclined to oppose euthanasia, viewing it as unnecessary in countries where it has been legalised.^35^ Van Hombeeck et al.^33^ found that Flemish nurses showed greater understanding of requests for euthanasia even in the absence of physical pain. In a recent study from Germany, the nurses emphasised their responsibility within interprofessional teams, and the important role that these teams have for discerning whether a patient’s request to die is genuinely a desire for death or an expression of a need for support in a challenging life situation.^34^

## Discussion

This scoping review identified several key findings regarding European nurses’ attitudes towards assisted death. First, nurses’ attitudes vary significantly across countries, influenced by legal frameworks, cultural norms and healthcare structures. Second, nurses expressed a strong desire for greater involvement in decision-making processes, particularly in countries where assisted death is legal. Third, ethical tensions were consistently reported, especially between respecting patient autonomy and adhering to the principle of non-maleficence. Finally, individual factors such as age, gender, religiosity, education, professional experience and work environment were found to shape nurses’ attitudes in nuanced ways.

This discussion addresses three key areas emerging from the review: (1) nurses’ involvement in assisted death decision-making, (2) ethical tensions between autonomy and non-maleficence and (3) contextual and experiential factors shaping attitudes. Rather than reiterating the findings, the discussion aims to interpret them in light of broader ethical and professional literature, including insights from a previous scoping review by Cayetano-Penman et al.^24^ which explored nurses’ perceptions of euthanasia globally. By comparing and contrasting our European findings with this earlier review, we seek to deepen the understanding of how nurses navigate the moral complexity of assisted death across diverse legal and cultural contexts. The discussion also integrates theoretical perspectives from care ethics and professional ethics to illuminate the normative implications of nurses’ attitudes and roles.

### Patients’ requests and nurses’ involvement

Across the included studies, nurses reported receiving requests for assisted death, even in countries where it is not legal. The line between a patient’s wish to not live and a formal request for assisted death is often subtle. Nurses are uniquely positioned to interpret these expressions, which may sometimes serve as emotional release. Verbalising such thoughts might help patients to process complex emotions, even in distressing circumstances. This underscores the need for nurses to have strong communication skills and ethical competence to navigate these sensitive conversations. Distinguishing genuine requests for assisted dying from expressions of suffering or depression is essential for delivering person-centred care.

The findings also reveal a strong desire among nurses for greater involvement in decision-making, particularly in countries where euthanasia is legal.^46,47^ As the largest healthcare profession and being closest to patients, nurses should be involved in decision-making at both macro and micro levels. This includes contributing to policy, legislation and ethical guidelines, as well as participating in clinical decisions. Their insights into patients’ needs and contexts are vital in interdisciplinary care. Greater involvement may also enhance nurses’ professional autonomy in a field that is traditionally dominated by physicians. However, their attitudes are often shaped by personal beliefs, possibly due to their historically limited role in decision-making. It may also reflect a lack of professional language to articulate ethical positions. As nurses take on more responsibility, it is crucial to remain aware of the tension between personal convictions and professional standards.

### Caught between conscience and code

This review highlights nurses’ varied levels of willingness to participate in euthanasia, which reflects internal ethical tensions between professional responsibilities and personal values. Attitudes towards assisted death were often shaped by personal beliefs, including religion and life philosophy. Nurses with strong religious convictions frequently opposed euthanasia, viewing life as sacred and divinely governed.^36,37,39,44,45,49^ Ethical frameworks, principles and guidelines also mattered, and those aligned with deontological ethics tended to reject euthanasia. This is noteworthy, as healthcare professionals are generally expected to act in accordance with shared professional standards rather than private convictions. According to Parsons’ classic theory of professions, nurses have a fiduciary duty to uphold competence and ethical standards,^50^ which includes adherence to professional codes of conduct.^51^ Yet, nurses’ motivations are not purely principled; they are also shaped by empathic responses to patient suffering.^23^ Their role morality thus exists in a tension between impartial ethical obligations and the personal domain of care.^23^ This duality was evident across the studies: nurses often referred to personal values when discussing assisted death, and some expressed willingness to participate if legalised, while others preferred to exclude certain tasks from their role. These differences suggest that nurses may experience role ambiguity when responding to patients requesting assisted death. In this context, democratic debate is essential, both in countries considering legalisation and those where it is under review. Nurses’ contributions to these debates should be grounded in professional reasoning, not solely personal belief. Empathy remains central to understanding patient suffering, often rooted in the thought that ‘it could have been me’.^52^ Given their close patient relationships, nurses are uniquely positioned to offer insights that can inform ethically sound and inclusive policy development. Nurses can therefore provide insights that enhance the quality and inclusivity of these debates, thereby helping to shape policies that are both ethically sound and practically effective.

Similar ethical tensions have been identified in previous research. For instance, Cayetano-Penman et al.^24^ found that nurses globally experience internal moral conflict and a sense of powerlessness when confronted with requests for euthanasia. These findings resonate with our review, which highlights the emotional and ethical burden nurses face, particularly when their professional responsibilities clash with personal convictions or institutional constraints.

### Between ethical principles and caring realities

There are notable differences in legal status and healthcare structures across the studied countries. These variations influence how assisted death is practiced and perceived, often shaping attitudes that may not reflect core ethical disagreements. Although such diversity can obscure the underlying ethical issues, several ethical challenges appear to be consistent across contexts. These include nurses’ struggles to balance respect for patient autonomy with the principle of non-maleficence, experiences of moral distress when confronted with requests for assisted death, and concerns about professional preparedness and ethical competence. Such recurring themes suggest that, despite legal and cultural differences, nurses across Europe face shared ethical tensions in their practice.

The review indicates that nurses across Europe face recurring ethical tensions in their practice, particularly when navigating requests for assisted death. These tensions often arise from the need to balance respect for patient autonomy with the principle of non-maleficence. Nurses may struggle to determine whether a request reflects a genuine desire to die or an expression of suffering that could be alleviated through care and support.

Principle-based ethics, which emphasise autonomy, beneficence and non-maleficence, offer one lens through^62^ which to understand these dilemmas. Nurses’ attitudes frequently reflect a conflict between respecting a patient’s right to choose and the professional obligation to do no harm. These tensions are especially acute in end-of-life care, where decisions carry profound moral weight – even when nurses are not the primary decision-makers or providers of assisted death.

At the same time, care ethics provides a complementary perspective, highlighting the relational and contextual nature of nursing practice. Virginia Held^53^ describes care ethics as a moral framework that arises in relationships among the unequal and dependent, where attentiveness, responsiveness and responsibility are central. This resonates with nurses’ particularistic reasoning, which often prioritises the needs of vulnerable individuals and resists rigid application of abstract principles.^23,54^ In situations involving assisted death, nurses may rely on relational understanding and empathy to guide their ethical judgement.

Together, these ethical frameworks illuminate the complexity of nurses’ moral reasoning and underscore the importance of ethical competence, reflective practice and interdisciplinary dialogue in managing assisted death requests.

### Shaping the attitudes: Contextual and experiential influences

Findings from several included studies indicate that education plays a pivotal role in shaping nurses’ attitudes towards euthanasia. Nurses with postgraduate training in palliative care often expressed less support,^32,34^ which is likely to be due to their emphasis on symptom management and holistic end-of-life care, which may reduce the perceived need for euthanasia. In contrast, those with broader academic exposure or specific ethics training may show more support, possibly due to increased engagement with complex moral reasoning.

The review found that nurses working in high-stress environments – such as night shifts, acute care units or wards with frequent end-of-life situations – were more likely to support euthanasia, suggesting that clinical context plays a critical role in shaping ethical perspectives.

Demographic factors, particularly age and gender, further shape attitudes, and younger nurses generally expressed more favourable views towards euthanasia and assisted suicide.^32,39,43,44,49^ While the included studies did not explore the influence of social media or societal trends directly, this generational pattern may correspond with broader public attitudes. This is also consistent with a supportive trend in the general population.^55^ However, support varies by type of assisted death; for example, younger nurses may be more supportive of physician-assisted suicide (PAS) than older colleagues, which indicate that attitudes are nuanced and context-dependent. The influence of demographic and professional factors is also supported by Cayetano-Penman et al.,^24^ who identified age, gender, religious beliefs and perceptions of palliative care quality as key determinants of nurses’ attitudes towards euthanasia. These factors align closely with our findings, suggesting that both personal and contextual elements shape nurses’ ethical positions across different settings.

As identified in the review, experienced nurses often express more context-sensitive attitudes towards assisted death. This may be explained by ethical theories that view experience as a moral resource. According to virtue-based and care-based ethical perspectives, experience plays a crucial role in moral development, as repeated engagement with ethically complex situations fosters practical wisdom, ethical sensitivity and relational understanding – all of which are essential for sound moral judgement.^56,57^ Additionally, the field of relational ethics highlights how close patient interactions shape nurses’ ethical judgements,^58^ whilst reflective equilibrium suggests that experience plays a crucial role in balancing ethical principles with the complexities of real-world situations.^59^ These perspectives help to explain why experienced nurses may develop more nuanced and context-sensitive attitudes towards assisted death.

### Ethical distinctions in attitudes: Between harm and relief

The review revealed that nurses’ attitudes towards euthanasia and assisted suicide are not always aligned, suggesting that ethical distinctions between actively causing death and allowing death to occur may influence their views. While both practices fall under the broader category of assisted death, some nurses appeared more accepting of physician-assisted suicide (PAS) than euthanasia, or vice versa. This divergence points to underlying moral reasoning that differentiates between ‘doing’ and ‘allowing’ harm.^60–61^ Note, however, that this distinction does not map neatly onto PAS and euthanasia in all cases.

Such distinctions are well established in ethical theory. From a consequentialist perspective, the moral weight turns on outcomes—so assisted suicide and euthanasia may be judged similarly when both relieve otherwise intractable suffering. By contrast, on deontological accounts there are stringent duties not to kill and to respect persons; hence actively causing death can be wrong regardless of outcomes, because it violates duties not to kill and to respect persons.

The ambiguity surrounding what constitutes harm or relief further complicates nurses’ ethical reasoning. If death is perceived as a harm, then any action contributing to it may be morally troubling. However, if death is seen as a release from unbearable suffering, then assisting in dying may be viewed as a compassionate and ethically justified act. This tension underscores the need for ethical reflection that is sensitive to context, patient experience and the relational nature of care.

By articulating these distinctions, nurses can better understand their own moral intuitions and contribute more effectively to interdisciplinary deliberations. Integrating ethical theory into professional education may also support nurses in navigating the complex moral terrain of end-of-life care.

This ethical ambiguity highlights the need for nuanced, empirically informed decision-making in nursing. Balancing patient autonomy with professional obligations requires careful ethical reflection, especially in end-of-life care. Both our review and that of Cayetano-Penman et al.^24^ emphasise the need for targeted education and clear professional guidelines to support nurses in ethically complex situations. Enhanced training in communication and ethical competence is essential to help nurses navigate requests for assisted death with confidence and clarity.

### Strengths and limitations

This scoping review has several strengths that enhance its credibility. It benefited from comprehensive database access and close collaboration with a university librarian, thereby ensuring a robust search strategy. Additionally, three researchers independently reviewed the articles, contributing diverse perspectives and minimising bias.

A key limitation is the conceptual inconsistency across included studies regarding terms such as ‘assisted death’, ‘euthanasia’, ‘medically assisted suicide’ (MAS) and ‘physician-assisted suicide’ (PAS), which complicated analysis and interpretation. A limitation of the search strategy is the inclusion of specific terms such as *attitudes* and *perceptions*, which may have narrowed the scope and excluded studies that address nurses’ views without explicitly using these terms. While these terms were included to ensure relevance to the research aim, a broader strategy – followed by manual assessment of attitudes during study selection – might have captured additional relevant literature. Further, the protocol was not registered, which may limit transparency. The exclusion of grey literature and non-English publications, and the absence of structured quality appraisal might limit the breadth and reliability of findings. Findings should be interpreted with caution due to the lack of formal quality appraisal, in line with scoping review methodology. Moreover, the use of varied, and in some cases untranslated, measurement instruments hindered consistent assessment of attitudes.

## Conclusion

This scoping review reveals the complex and varied attitudes of European nurses towards assisted death, which are shaped by legal, cultural and healthcare differences. Nurses face ethical tensions between care-based and principle-based approaches, particularly between respecting autonomy and avoiding harm. These challenges are compounded by the need to balance personal beliefs with professional responsibilities. The findings highlight the importance of ethical competence, communication skills and nurse involvement in both policy and clinical decision-making. Despite some limitations of this scoping review, it highlights critical areas for future research, including the need for more consistent definitions of assisted death, greater exploration of nurses’ roles in interdisciplinary teams and the development of targeted education and training programmes.

## Data Availability

This scoping review is based on publicly available literature. No new data were generated or analysed during this study. Therefore, data sharing is not applicable to this article.*
